# Red and Green Algal Origin of Diatom Membrane Transporters: Insights into Environmental Adaptation and Cell Evolution

**DOI:** 10.1371/journal.pone.0029138

**Published:** 2011-12-14

**Authors:** Cheong Xin Chan, Adrian Reyes-Prieto, Debashish Bhattacharya

**Affiliations:** Department of Ecology, Evolution and Natural Resources and Institute of Marine and Coastal Sciences, Rutgers University, New Brunswick, New Jersey, United States of America; University of Melbourne, Australia

## Abstract

Membrane transporters (MTs) facilitate the movement of molecules between cellular compartments. The evolutionary history of these key components of eukaryote genomes remains unclear. Many photosynthetic microbial eukaryotes (*e.g.*, diatoms, haptophytes, and dinoflagellates) appear to have undergone serial endosymbiosis and thereby recruited foreign genes through endosymbiotic/horizontal gene transfer (E/HGT). Here we used the diatoms *Thalassiosira pseudonana* and *Phaeodactylum tricornutum* as models to examine the evolutionary origin of MTs in this important group of marine primary producers. Using phylogenomics, we used 1,014 diatom MTs as query against a broadly sampled protein sequence database that includes novel genome data from the mesophilic red algae *Porphyridium cruentum* and *Calliarthron tuberculosum*, and the stramenopile *Ectocarpus siliculosus*. Our conservative approach resulted in 879 maximum likelihood trees of which 399 genes show a non-lineal history between diatoms and other eukaryotes and prokaryotes (at the bootstrap value ≥70%). Of the eukaryote-derived MTs, 172 (ca. 25% of 697 examined phylogenies) have members of both red/green algae as sister groups, with 103 putatively arising from green algae, 19 from red algae, and 50 have an unresolved affiliation to red and/or green algae. We used topology tests to analyze the most convincing cases of non-lineal gene history in which red and/or green algae were nested within stramenopiles. This analysis showed that ca. 6% of all trees (our most conservative estimate) support an algal origin of MTs in stramenopiles with the majority derived from green algae. Our findings demonstrate the complex evolutionary history of photosynthetic eukaryotes and indicate a reticulate origin of MT genes in diatoms. We postulate that the algal-derived MTs acquired via E/HGT provided diatoms and other related microbial eukaryotes the ability to persist under conditions of fluctuating ocean chemistry, likely contributing to their great success in marine environments.

## Introduction

Membrane transporters (MTs) are a large, structurally and functionally heterogeneous group of proteins; *i.e.*, carriers and channels [1] that span cell lipid bilayers. These proteins mediate or facilitate the transfer of ions and molecules across biomembranes. MTs are essential for nutrient (*e.g.*, amino acids and sugars) uptake, extrusion of harmful chemicals (*e.g.*, toxins and heavy metals), and compartmentalization *via* generation of transmembrane ionic concentration gradients (*e.g.*, Ca^2+^, K^+^, Na^+^, H^+^). The variety and the number of MTs expressed in a particular cell (*i.e.*, the permeome) directly reflect the metabolic/physiological flexibility and lifestyle of the organism [2]–[5]. For example, genomes of heterotrophic and mixotrophic organisms encode numerous MTs for exogenous nutrient uptake, whereas genomes of metal tolerant algae contain numerous metal transporters that play essential roles in detoxification [2]. The biochemistry and mode of action of many MTs are well understood. In contrast, little is known about their evolutionary history in eukaryote lineages.

Genome evolution of eukaryotes is implicated by multiple instances of foreign gene acquisition *via* horizontal gene transfer (HGT), particularly among unicellular taxa such as diatoms, excavates, dinoflagellates, ciliates, and chlorarachniophytes [6]–[10]. In addition, algal groups bearing plastids (*e.g.*, Plantae, diatoms) have recruited numerous foreign sequences *via* endosymbiotic gene transfer (EGT). The latter is a special case of HGT whereby genes are transferred to the host nucleus from permanent intracellular endosymbionts [8], [11], [12]. Because the genomes of the two diatom species *Thalassiosira pseudonana* and *Phaeodactylum tricornutum* have been sequenced to completion and well annotated [13], [14], they provide a promising subject for assessing the evolutionary history of MTs in microbial eukaryotes.

In an earlier genome-wide study [15], 600–700 phylogenies of diatom proteins were found to provide robust support for a specific affiliation between diatoms and other lineages that often include the chlorophyll *a* + *c*-containing eukaryotes (*e.g.*, photosynthetic stramenopiles, dinoflagellates, haptophytes, cryptophytes; often loosely grouped together as the “chromalveolates” [16]) and prasinophyte green algae. This finding suggested that many prasinophyte-related genes may have arisen *via* EGT from an ancient, cryptic (plastid) endosymbiosis that occurred before the capture of the widely distributed red algal plastid in photosynthetic chromalveolates [17], [18]. The original chromalveolate hypothesis [16] envisioned a single origin of the red algal derived plastid in all chromalveolates. This idea was falsified in recent studies that suggest the involvement of other [15], [19] or recurrent [20] eukaryote endosymbiosis during chromalveolate evolution, as well as a different phylogenetic relationship among these taxa than originally proposed. The findings of these studies most certainly reflect more than stochastic mutation rate variation in the sequences, which could have introduced biases in phylogeny inference. However, the overall monophyly of chromalveolate host taxa (*i.e.*, independent of their plastid history) remains a testable, albeit challenging working hypothesis in phylogenetics.

In spite of these issues, a recent phylogenomic analysis of the brown seaweed *Ectocarpus siliculosus* [21] revealed 611 genes of putative red algal origin (189 encoding putative plastid-targeted proteins [PPTPs]) and 2,669 genes of likely green algal provenance (67 encoding PPTPs). These results clearly demonstrate the existence of a green algal (hereinafter, green) “footprint” that is robustly recovered (minimally) in the common ancestor of brown algae and diatoms (*i.e.,* Ochrophyta [22]). An alternative explanation for the finding of green genes in diatoms, whether they arose *via* EGT or HGT, is incomplete taxon sampling in many trees that misleads phylogenetic inference of gene origin. It is however noteworthy that the putative green genes reported in chromalveolates are distributed across diverse cellular processes and not restricted to the plastid proteome [15], [21]. This underscores the importance of categorizing the functions and physiological roles of these foreign sequences to understand the biological consequences of widespread gene transfer.

Here we examined the evolutionary history of 1,014 bioinformatically predicted MTs in the diatoms *T. pseudonana* and *P. tricornutum* that are available at TransportDB (http://www.membranetransport.org/; [24]). Previous genome studies in diatoms indicated the presence of multiple MTs for inorganic nutrients such as ammonium, nitrate, phosphate, sulfate, bicarbonate, silicic acid, and as well for urea, amino acids, and sugars [13], [14]. We assessed individual phylogenies for each of the predicted MTs to determine the proportion of the diatom permeome that originated *via* HGT/EGT from red and green algal sources. The limited taxon sampling of microbial eukaryotes, particularly of the mesophilic red algae is a key limiting factor in phylogenetic analysis involving these organisms, as demonstrated in previous studies [10], [15], [23]. The only red algal genome data available to these studies were from the thermoacidophilic Cyanidiales [25] that possess highly reduced and specialized genomes [2]. Genome size and gene content in Cyanidiales (*e.g.*, 16.6 Mbp genome of *Cyanidioschyzon merolae* encoding 5,331 genes [25]) contrasts strongly to that of mesophilic green algae such as the model species *Chlamydomonas reinhardtii* (121 Mbp genome encoding ca. 15,143 genes [26]). The impact of using data solely from highly reduced red algal genomes is that homologous gene copies present in mesophilic rhodophytes will appear, by virtue of their absence in the database, to be of green algal descent. To address this key issue in phylogenomics we included in our analysis partial genome data from the mesophilic red algae *Porphyridium cruentum* (unicellular bangiophyte) and *Calliarthron tuberculosum* (multicellular, coralline florideophyte) that were recently published [27], as well as the recently published data from the photosynthetic, multicellular stramenopile *Ectocarpus siliculosus* [21].

## Results and Discussion

We retrieved 1,014 MTs from *P. tricornutum* (514) and *T. pseudonana* (500) that are available at TransportDB [24]. These provide a set of well-annotated, unique proteins that have been classified into various MT families in TransportDB based on their putative functions (see Table S1). We used these proteins to elucidate their evolutionary histories using a phylogenomic approach. Here we define a taxon as each individual terminal node of a phylogenetic tree and a phylum as the group of such closely related lineages such as the stramenopiles, Alveolata, Rhodophyta, and Viridiplantae. We infer a non-lineal gene history (*i.e.*, not sharing a single common ancestor) when a well-supported node (*i.e.,* bootstrap cut-off value ≥70%) is recovered that unites members of two phyla regardless of the direction of gene transfer between them.

### Exclusive phyletic association of diatom MT genes

For each of the diatom MT proteins, we first used a simplified reciprocal BLAST approach [27] to identify putative homologs in other taxa (based on sequence similarity), using a broadly sampled protein database that consists of ca. 15 million sequences (see Materials and Methods, and Table S2). We found 31 (3.1%) of the 1,014 diatom MTs to be highly similar to proteins in only one other taxon in the database (BLASTP [28], *e*-value ≤10^−10^). These proteins presumably indicate a close evolutionary relationship between the two phyla. Similar to an earlier study [27], we examined the two-phylum associations across an increasing number of hits per query (*x*) from both phyla. Our assumption was that systematic bias introduced by inadequate taxon sampling decreased as the total number of hits (*i.e.*, taxonomic breadth) increased. Figure S1 shows the distribution of the diatom MT proteins that exhibit a strong association with one other phylum at *x* ≥2, ≥10, and ≥20. At *x* ≥2 (Figure S1A), diatom MT proteins showed a strong association with stramenopiles (13), Haptophyta (5), Alveolata (4), Fungi (2), and various other phyla. Among the seven MTs at *x* ≥10 (Figure S1B), a majority had hits only to stramenopiles (5), when compared to Fungi (1) and Metazoa (1). In contrast, two of the three diatom MTs for which ≥20 hits were found showed a strong association with the stramenopiles, and one with Metazoa (Figure S1C). As *x* increases from ≥2, ≥10 to ≥20, we observe a general decrease in the proportions of Haptophyta (16 → 0 → 0%), Alveolata (13 → 0 → 0%), and Fungi (6 → 14 → 0). Therefore, the association between diatoms and these “foreign” taxa are likely due to imbalanced taxon sampling in the current database; *e.g.*, proteins from Fungi and Metazoa constitute ca. 30% of the current database, compared to the limited data that is available from microbial eukaryotes (see Table S2). This is in contrast to the increasing proportions of proteins associated with stramenopiles (42 → 71 → 67%). Although these 31 proteins represent a relatively small proportion of diatom MTs, our observations suggest a signal of evolutionary association for diatom MTs with the stramenopiles, as expected under vertical gene inheritance.

### Evolutionary origin of diatom MTs

Next we used phylogenomics to assess the evolutionary origins of the remaining MTs in diatoms. We generated a maximum likelihood [29] phylogeny for each of the 879 MT protein alignments in which an alignment contained ≥4 sequences and ≥50 aligned amino acid positions after removal of divergent, ambiguously aligned blocks (see Materials and Methods). To further minimize the impact of phylogenetic artifacts (*e.g.*, inadequate taxon sampling and stochastic sequence variations) [27], we restricted our analysis to 697 trees that each contained ≥3 distinct phyla and ≥30 terminal taxa. To infer the evolutionary history of these proteins, we adopted a two-step approach of phylogeny sorting, in which we first used a simple computational approach to rapidly identify trees with potentially (interpretable) topologies, that was followed by manual inspection of each tree (see Materials and Methods). The first step identified 399 phylogenies at bootstrap ≥70% (321 at bootstrap ≥90%) that fit our criteria; *i.e.*, these phylogenies (ca. 37–45% of the overall 879 phylogenies) show a reasonably strong signal for inferring the evolutionary origin of diatom MT genes.

**Figure 1:**
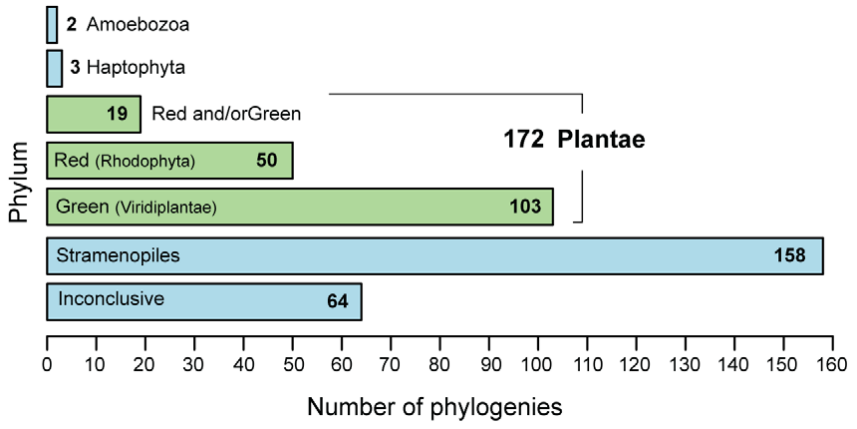
Putative origin of membrane transporter genes in diatoms. The Plantae lineages, *i.e.*, the red algae (Rhodophyta) and the green algae/plants (Viridiplantae), and the red and/or green algae (Red+Green) are highlighted in green. Number of phylogenies supporting monophyly (bootstrap ≥70%) between diatoms and each phylum is shown in each bar. The 473 trees shown are a result of our two-phase approach for phylogeny sorting upon examination of 851 trees generated in this study, in which each tree contains ≥3 distinct phyla and ≥30 terminal taxa.

The results of the manual inspection of the 399 phylogenies are shown in Table S3. We found 172 (24.6% of the total 697 examined phylogenies) to provide evidence of a non-lineal gene history in diatoms with the red and/or green algae, 158 (22.7%) with stramenopiles, 3 (0.4%) with haptophytes, and 2 (0.3%) with Amoebozoa. Gene origin in the remaining trees is inconclusive (Figure 1). Whereas the strong association between diatoms and stramenopiles (and Apicomplexa, under the SAR hypothesis which also includes the Rhizaria [19], [30]) in the 158 MT genes can be explained by vertical inheritance, our results suggest that at least 177 (25.3%) of the 679 examined phylogenies of diatom MTs are likely to have undergone E/HGT with other lineages of eukaryotes. Alternatively, these observations (*e.g.*, the small number of MTs showing evidence of non-lineal association with haptophytes, and Amoebozoa) can be explained by gene loss in other closely related lineages of diatoms such as the stramenopiles and alveolates, or by an imbalance in taxon sampling in the current database. Nevertheless, the strong connection we observed between the diatoms and the red/green algae is likely to be explained by HGT related to an endosymbiotic association of the algal lineages; *i.e.*, EGT rather than due to phylogenetic artifacts. The summary of all 1,014 diatom MTs used in this study, and their putative partners of E/HGT are shown in Table S4. The majority of these proteins have an unresolved phylogenetic history using our approach that may reflect the limitation of currently available genome data. Nevertheless, this result indicates the complexity of evolutionary history of diatoms and other microbial eukaryotes, as has been shown in other genome-wide studies [20], [27].

### Putative E/HGT of transporter-encoding genes between diatom and red/green algal lineages

The inference of gene origin based on phylogenetic relationships can vary depending on the position at which the tree is rooted with a specified outgroup. Here we used prokaryote lineages as outgroups where available, and in their absence the Opisthokonta (Metazoa or Fungi) were used to root the trees. Given the large amount of prokaryote data in the database (62% of the total 15.2 million sequences; Table S2) and assuming an ancient divergence between prokaryotes and eukaryotes [31], the presence of the more distantly related prokaryote MTs suggests that most eukaryote genes that are currently available would have been recovered in our analysis (*i.e.,* under the cut-offs used). This of course does not prevent under-sampling eukaryote MT diversity, in particular if many of these genes are highly divergent and not identified as homologs or are missing from partial EST datasets. Overall, 131 (76%) of 172 algal derived MTs in diatoms have prokaryote homologs that were used as outgroups to root these trees (Table S3).

Of the 172 algal derived MTs, 103 are associated with green algae, 19 with red algae, 50 with red and/or green algae. These MTs can be further clustered into protein families of similar function based on overlapping sequences among the individual phylogenies (see Materials and Methods). These clusters do not represent sets of well-defined paralogous protein families, but simply represent phylogenies consisting overlapping sequences in a gene-by-gene survey, which display similar topologies. Table 1 shows the clusters of putative green algal derived MTs, and Table 2 shows the protein clusters that are putatively derived from the red and/or green algae. Using a combinatorial approach for predicting subcellular localization of stramenopile (heterokont) proteins as implemented in HECTAR [32], we assessed the putative targets of these diatom MTs based on the presence of signal peptide, transit peptide and/or a type II signal anchor in the sequences (see Materials and Methods). Of the 39 green algal derived protein clusters (Table 1), two and one show evidence of plastid- and mitochondrion-targeting, respectively. The detection of a signal peptide in 10/39 clusters suggests these proteins are likely to be membrane-associated. In comparison, 5/28 protein clusters in Table 2 show evidence of plastid-targeting (an additional two of mitochondrion-targeting), and a signal peptide was detected in 8 clusters. The plastid-targeted, red algal derived proteins can be explained by the secondary endosymbiosis that gave rise to the diatom plastid; *i.e.*, EGT from the nucleus of the red algal endosymbiont to support organelle functions [33]. The presence of a signal anchor in 13 protein clusters shown in both Tables 1 and 2 indicates putative transmembrane protein/channel proteins, whereas the majority lacks a N-terminal targeting sequence. Therefore, the algal-derived, nuclear-encoded MTs in diatoms likely extend beyond plastid and photosynthetic functions.

**Table 1:** The 103 putatively green algal derived MTs in diatoms, clustered based on similar functions.

Cluster ID	Size	Putative function	MT family	Outgroup	HECTAR output
A02NE20	2	MATE efflux family protein chloroplastic	MOP	Prokaryote	-
A02NE21	1	Mitochondrial substrate carrier family protein N	MC	Metazoa	-
A03EX01	2	Magnesium transporter MgtE	MgtE	Prokaryote	SA
A03EX02	6	MATE efflux family protein chloroplastic	MOP	Prokaryote	CHL
A03EX03	3	Mitochondrial substrate carrier family proteins	MC	Prokaryote	-
A03EX04	3	Cadmium- or zinc-transporting ATPase	P-ATPase	Prokaryote	-
A03EX05	4	Sulfur deprivation response regulator	DASS	Prokaryote	SP
A03EX06	1	Sodium/calcium exchanger protein	CaCA	Metazoa	SA
A03NE02	1	Adenine guanine permease	NCS2	Prokaryote	-
A03NE03	6	Solute carrier family 35 member B1	DMT	Metazoa	SP
A03NE06	2	Zinc transporter	ZIP	Prokaryote	SP
A03NE07	2	Calcium-binding mitochondrial carrier protein	MC	Metazoa	-
A03NE08	5	Transmembrane amino acid transporter	APC	Prokaryote	SP
A03NE10	1	Multidrug resistance-associated proteins	ABC	Metazoa	-
A03NE12	7	D-xylose-proton symporter-like 1/sugar transporter	MFS	Prokaryote	SP
A03NE13	2	Transporter ArsB	ArsB	Prokaryote	-
A03NE16	11	MATE efflux family protein chloroplastic	MOP	Prokaryote	MIT
A03NE17	1	Eukaryotic translation initiation factor 4 gamma 3	MFS	Prokaryote	-
A03NE18	1	Copper-transporting ATPase 3	P-ATPase	Prokaryote	-
A03NE19	1	Calcium-transporting ATPase PAT1	P-ATPase	Metazoa	-
A03NE21	4	Adenosine 3-phospho 5-phosphosulfate transporter 2	DMT	Metazoa	SA
A03NE22	2	Phosphate-binding protein PstS	ABC	Prokaryote	SP
A03NE24	3	SPX domain-containing membrane protein	MFS	Fungi	-
A03NE25	2	Uncharacterized protein C1orf53	ABC	Prokaryote	-
A03NE29	2	Synaptic vesicle 2-related protein	MFS	Prokaryote	SP
A03NE31	1	Vacuolar cation proton exchanger 5	CaCA	Prokaryote	-
A03NE32	2	Uncharacterized membrane protein STKORF319	TerC	Prokaryote	SP
A03NE33	3	Sodium bile acid cotransporter 7	BASS	Prokaryote	-
A03NE35	2	Glutathione S-transferase	CLIC	Metazoa	CHL
A03NE37	2	Probable sugar phosphate phosphate translocator At1g06470	DMT	Metazoa	-
A03NE38	4	Calcium-transporting endoplasmic reticulum-type	P-ATPase	Prokaryote	SA
A03NE39	2	K^+^-stimulated pyrophosphate-energized sodium pump	H+-PPase	Prokaryote	SA
A03NE40	3	ABC transporter G family members	ABC	Metazoa	-
A03NE41	2	Solute carrier family 25 members	MC	Metazoa	SP
A03NE44	1	ABC transporter B family member 1	ABC	Prokaryote	-
A03NE45	1	Two pore calcium channel protein 1	VIC	Metazoa	SA
A03NE46	1	Sodium:solute symporter family	SSS	Prokaryote	SA
A03NE47	1	Sulfate transporter YbaR	SulP	Prokaryote	SP
A18NE06	3	Probable cation-transporting ATPase F	P-ATPase	Prokaryote	SA

Note: Shown for each cluster is the identifier (Cluster ID), number of proteins within the cluster (Size), the putative function, the classification of MT family based on TransportDB, the outgroup used in phylogeny sorting, and the protein target prediction using HECTAR, in which “CHL” denotes proteins targeting to chloroplast/plastid, “MIT” denotes proteins targeting to mitochondrion, “SP” denotes presence of signal peptide, “SA” denotes presence of Type II signal anchor, “-” denotes no N-terminal target peptide found. None of these proteins show evidence of plastid- or mitochondrion-targeting. The abbreviation of MT family follows Table S1, according to http://www.membranetransport.org/.

**Table 2:** The 69 diatom MTs that are putatively derived from red algae, and red and/or green algae, clustered based on similar functions.

Cluster ID	Size	Putative function	MT family	Outgroup	Algal origin	HECTAR output
A02NE02	2	Protein translocase subunit secA	IISP	Prokaryote	R	-
A02NE04	1	Sodium bicarbonate cotransporter 3	AE	Prokaryote	R	CHL
A02NE06	9	High-affinity nitrate transporter	MFS	Prokaryote	R	SA
A02NE19	2	Magnesium and cobalt efflux protein	HCC	Prokaryote	R	SP
A03NE28	2	Signal sequence-binding protein	IISP	Prokaryote	R	-
A04NE01	1	S-adenosylmethionine mitochondrial carrier protein	MC	Metazoa	R	-
A04NE02	1	Solute carrier family 25 member 36	MC	Metazoa	R	-
A04NE03	1	HCO_3_^-^ transporter family	AE	Metazoa	R	SA
A02NE09	3	Uncharacterized sodium-dependent transporter	BASS	Prokaryote	R+G	SP
A03NE27	3	K^+^ efflux antiporter chloroplastic	CPA2	Prokaryote	R+G	CHL
A02NE01	1	Adenosine 3-phospho 5-phosphosulfate transporter 1	DMT	Metazoa	R/G	-
A02NE05	3	Chloride channel protein CLC-f	ClC	Prokaryote	R/G	MIT
A02NE07	4	Inner membrane protein chloroplastic	Oxa1	Prokaryote	R/G	CHL
A02NE10	1	Protein grpE	MPT	Prokaryote	R/G	-
A02NE11	2	S-adenosylmethionine mitochondrial carrier protein	MC	Fungi	R/G	SP
A02NE12	1	Glutathione S-transferase DHAR2	CLIC	Prokaryote	R/G	SA
A02NE13	2	Low affinity tryptophan permease	HAAAP	Prokaryote	R/G	SA
A02NE14	1	Sodium-dependent phosphate transport protein chloroplastic	MFS	Prokaryote	R/G	SP
A02NE15	5	Folate/biopterin transporter	FBT	Prokaryote	R/G	SA
A02NE16	2	YggT/hypothetical protein	YggT	Prokaryote	R/G	SP
A02NE17	4	ABC transporter D family member chloroplastic	ABC	Prokaryote	R/G	MIT
A02NE18	2	ABC transporter G family member 7	ABC	Metazoa	R/G	SP
A02NE22	1	ATP synthase gamma chloroplastic	F-ATPase	Prokaryote	R/G	CHL
A03NE14	1	Anion exchanger	AE	Metazoa	R/G	-
A03NE23	3	Sodium bile acid cotransporter	BASS	Prokaryote	R/G	SP
A03NE26	3	Permease of the drug/metabolite transporter	DMT	Prokaryote	R/G	CHL
A03NE36	5	ABC transporter ATP-binding protein	ABC	Prokaryote	R/G	-
A03NE48	3	K^+^ efflux antiporter chloroplastic	CPA2	Prokaryote	R/G	SP

Shown for each cluster is the identifier (Cluster ID), number of proteins within the cluster (Size), the putative function, the classification of MT family based on TransportDB, the outgroup used in phylogeny sorting, the putative algal origin of the proteins, *i.e.*, Red (R), Red or Green (R/G), and Red and Green (R+G), and the protein target prediction using HECTAR, in which “CHL” denotes proteins targeting to chloroplast/plastid, “MIT” denotes proteins targeting to mitochondrion, “SP” denotes presence of signal peptide, “SA” denotes presence of Type II signal anchor, “-” denotes no N-terminal target peptide found. None of these proteins show evidence of mitochondrion-targeting. The abbreviation of MT family follows Table S1, according to http://www.membranetransport.org/.

**Figure 2:**
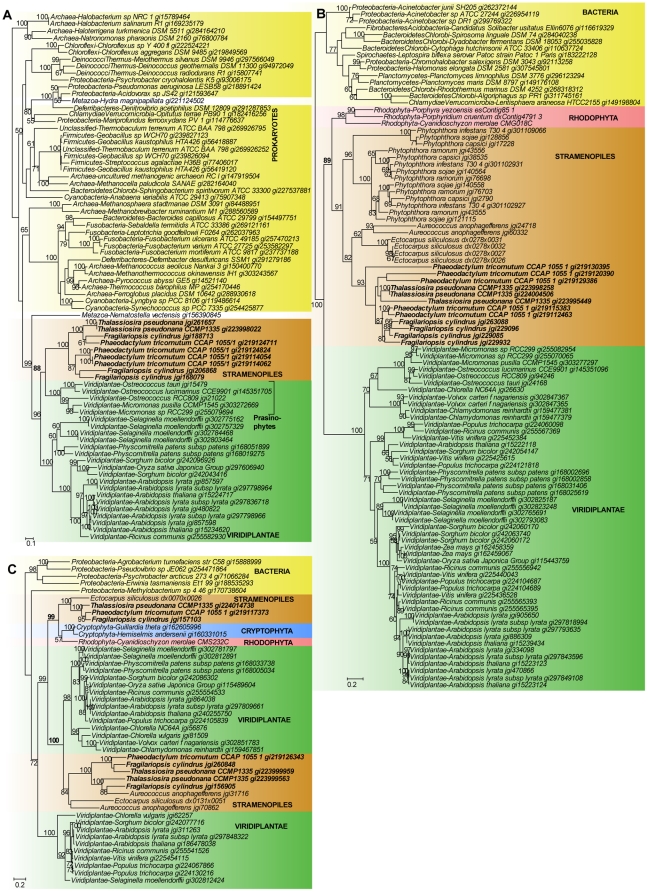
Diatom membrane transporters of putative algal origin. The phylogenies shown are of (A) a green algal derived zinc/cadmium/cobalt transporter, (B) a red algal derived high affinity nitrate transporter, and (C) a red and green algal derived potassium efflux antiporter. In all trees node values represent RAxML bootstrap support for 100 subsamples. Prokaryotes are highlighted in yellow, Stramenopiles in brown, and Cryptophyta in blue. The Plantae lineages, Rhodophyta and Viridiplantae are highlighted in red and green, respectively. Diatom species are shown in boldface. Only bootstrap values ≥50% are shown. The unit of branch length is the number of substitutions per site.

As shown in Table 1, the majority of green algal derived MTs in diatoms have putative functions related to multidrug/oligosaccharidyl-lipid/polysaccharide flippase family (MOP; 19 MTs in 3 clusters), the major facilitator superfamily (MFS; 13 in 4 clusters), drug/metabolite transporters (DMT; 12 in 3 clusters), and the P-type ATPase (P-ATPase; 12 in 5 clusters). These MTs include a number of transporters for metal cations; *e.g.*, sodium, potassium, calcium, cadmium, magnesium, and zinc, with diverse mechanisms of action such as anion exchangers, symporters, and translocators. Figure 2A shows a phylogeny of a protein encoding a zinc/cadmium/cobalt transporter in diatoms that is putatively derived from prasinophyte green algae. The phylogeny shows a strong monophyletic support (bootstrap 88%) between lineages of diatoms and prasinophytes, whereas the other green algae and plants (Viridiplantae) branch as sister to this group within a strongly supported clade (bootstrap 96%). This observation is incongruent with the Plantae hypothesis (that groups red, green and glaucophyte algae in one clade [34], [35]), or the well-supported monophyly of Viridiplantae [36], therefore a close connection between diatoms and prasinophytes to the exclusion of other green lineages likely reflects non-lineal history rather than a vertical relationship. The large number of green algal derived genes may be explained by a cryptic endosymbiotic association between diatoms and prasinophytes, in agreement with a previous genome-wide phylogenomic analysis of diatoms [15]. The remaining green algal set identified in our analysis may include genes that were derived from such a cryptic endosymbiotic association, but have been lost from the reduced genomes of our reference picoprasinophyte taxa [15], [37].

In contrast, the majority of red algal derived MTs in diatoms (Table 2) have functions related to MFS (9 in 1 cluster), the type II (general) secretory pathway (IISP; 4 in 2 clusters), mitochondrial carriers (MC; 2 in 2 clusters), anion exchangers (AE; 2 in 2 clusters), and the HlyC/CorC family (HCC, related to cobalt-resistance; 2 in 1 cluster). Notably, these MTs include high-affinity nitrate transporters (9). The phylogeny of the bacterial derived high-affinity nitrate transporter in diatoms is shown in Figure 2B, that shows strongly the supported monophyly (bootstrap 89%) of diatoms (including other stramenopiles) and red algae. This clade is sister to Viridiplantae at bootstrap 100%. This topology suggests the transfer of red algal genes into the ancient lineage leading to all stramenopiles. A putative (although unspecified) algal origin of nitrate transporters in diatoms and the closely related lineages of dinoflagellates have previously been described [38], [39]. With the inclusion of a rich red algal gene repertoire (*e.g.*, from *Porphyridium cruentum*) here we found evidence of a red algal origin of all 9 high-affinity nitrate transporters in diatoms (Table S4) and in stramenopiles.

Whereas the potential red/green origin of 43 algal derived MTs in diatoms could not be unambiguously determined (Table 2), we found two MT clusters to show evidence of both red and green algal origin, the putative sodium-dependent transporters (3) and potassium efflux antiporters (3). Figure 2C shows the phylogeny of a potassium efflux antiporter in diatoms, in which two independent monophyletic groups involving the diatom lineages are observed: one clade (bootstrap 100%) consists of sequences from *P. tricornutum* and *T. pseudonana*, the red alga *C. merolae* and cryptophytes (*i.e.*, the genes from diatoms and cryptophytes are likely red algal derived), and another (bootstrap 99%) consists of genes from diatoms (as well as other stramenopiles including *Ectocarpus siliculosus*) and green algae, suggesting a green algal origin. The other green algae and plants are grouped in a separate clade (bootstrap 100%) elsewhere in the tree, suggesting the strong association of diatom lineages with red and green algae is likely due to E/HGT. Alternatively, this phylogeny may be explained by a single common origin in the ancestor of eukaryotes followed by massive loss with only Plantae and “chromalveolates” retaining the gene. In either case, this nuclear-encoded protein in diatoms is plastid-targeted, indicating a possible linkage to photosynthetic function and has an endosymbiotic provenance involving the red and/or green algae.

### Assessment of algal E/HGT in stramenopiles

Our analysis thus far assumes that a strongly supported monophyletic grouping of diatoms and distantly related algae implies a close association between the two phyla, and therefore E/HGT. However, these results could also be explained by insufficient sampling of stramenopiles in the database, convergent evolution among these lineages, the less-plausible explanation of massive gene loss across other eukaryote lineages, or simply phylogenetic artifacts that have misled the data interpretation. To gain another perspective on the impact of algal gene transfer on diatom MT evolution, we explored the extent of algal E/HGT in all stramenopiles in the MT trees rather than just focusing on diatoms. Among the 697 protein phylogenies considered (each contained ≥3 distinct phyla and ≥30 terminal taxa), we identified 75 that contain one or more strongly supported monophyletic clades (bootstrap ≥70%) grouping the stramenopiles and Plantae lineages (11 with Rhodophyta, 55 with Viridiplantae, and 9 with both lineages). For each of these 75 trees, we examined the fit of the data (*i.e.,* each protein alignment) that gave rise to the RAxML “best” tree, when compared to a null hypothesis of a monophyletic stramenopile clade that is not interrupted by (*i.e.*, have a nested relationship with) red/green algae. The latter was generated from the corresponding RAxML trees by moving the red/green algae outside the stramenopiles. Here we did not consider gene transfers involving other taxa and did not change any other branching positions in the trees. For each instance, the RAxML tree was compared to the rearranged (reference) topology using the one-sided KH test [40], the SH test [41], the test of expected likelihood weights (ELW) [42], and the two-sided (original) KH test [43], as implemented in Tree-Puzzle 5.2 [44]. Significant rejection of the null hypothesis [e.g., 45] for each test required a *p*-value ≤0.05.

Of the 75 MT trees analyzed using this approach, 58 (77.3%) indicate that the RAxML tree has a better fit to the data than the reference topology (compared to 17 that favor the reference tree), of which 8 (10.7%) show a significant rejection of the null hypothesis across all four statistical tests (Table S5). Two other MT trees show rejection in one and two statistical tests, respectively, whereas 48 do not significantly reject the null hypothesis. None of the rearranged trees were significantly better than the RAxML best tree. Interestingly, 55 (73.3%) of the 75 MTs were identified as putatively red and/or green algal derived in diatoms based on our phylogenomic analysis (35 from green algae, 12 from red algae, 8 from red and/or green algae; Table S5). An additional five were inferred as stramenopile-derived, whereas the remaining 15 yielded unresolved origins in diatoms. Of these 55 trees, 14 show the reference topology to be a better fit to the data than the RAxML tree, suggesting these are not instances of E/HGT, at least in terms of the stramenopile clade. In contrast, the remaining 41 trees suggest E/HGT between stramenopiles and the algal lineages (4 with Rhodophyta, 29 with Viridiplantae, and 8 with Rhodophyta and/or Viridiplantae). Therefore in these instances red/green algal MT origin in diatoms is likely. The origin of these MTs in diatoms was identified as red algae (10), green algae (26), and red and/or green algae (5) based on our phylogenomic approach. In particular, the eight trees that show significant rejection of null hypothesis across all four tests represent highly likely cases of E/HGT involving stramenopiles and Viridiplantae (with one exception between stramenopiles and both Rhodophyta and Viridiplantae). These proteins represent the most conservative estimate of algal derived MTs in diatoms. In summary, our data suggest that 8 (0.1%) to 58 (8.3%) of the 697 examined topologies show potential E/HGT between stramenopiles and Plantae lineages. When considering both the phylogenomic and the topology test approach, we estimate the red/green algal contribution to the diatom MT repertoire ranges from the most conservative estimate of 8 (0.1% of examined phylogenies) to 41 (5.9%) in which strong evidence of a E/HGT history involving stramenopiles and Plantae lineages is required, to 172 (24.7%) using our phylogenomic approach that is based on our two-phase inspection of strongly supported monophyletic clades specifically between diatoms and Plantae.

### Acquisition of key algal derived MT genes in diatoms

Our findings suggest that algal derived MTs in diatoms are involved in the movement of metal cations (especially among the green algal derived MTs), extending beyond plastid and photosynthetic functions, as shown in Tables 1 and 2. Metal cations are essential cofactors of several metalloproteins involved in plastid functions [3] such as photosystems and plastocyanins [46] and others including coenzyme A synthases and superoxide dismutases. The maintenance of intracellular metal balance is an essential cellular process, because excessive accumulation of heavy metals can be toxic for the cell. Given the highly specific and obligate requirement for metals, free-living unicellular algae possess efficient transport systems for both metal uptake and detoxification (see [4]) to regulate metal homeostasis [3]. Therefore, under the scenario of a cryptic green algal endosymbiosis, we suggest that the diatom ancestor (and likely other stramenopiles) benefited from this ancient endosymbiosis by not only gaining photosynthetic capacity for the host cell *via* the captured plastid but also access to proteins for cell detoxification *via* EGT from the captured nucleus. Photosynthetic cells that inhabit aquatic environments are known to have efficient mechanisms to cope with major fluctuations in nutrient availability, light intensity, and other abiotic conditions (*e.g.,* [47]). Genome analyses of marine photosynthetic cells [48] reveal genes encoding numerous metal transporters and metalloenzymes that are likely involved in the maintenance of cell fitness in coastal waters.

Potassium and sodium are important monovalent cations that are involved in many biological processes such as enzyme activation, regulation of osmotic pressure and membrane electric potential, as well as growth and development. Potassium transporters are known in some instances to be reactive to sodium ions (that are abundant in the marine environment), given the highly similar chemical structures shared by the two cations [49]. Therefore, the acquisition and retention of exogenous genes related to potassium/sodium transport functions, particularly from both sources of red and green algal lineages (Figure 2C and Table 2), may reflect the crucial ecophysiological adaption of ancestral phytoplankton lineages (including diatoms) to the interchange between freshwater and marine conditions (*e.g.*, [2]), to ensure cell survival. Adaptive evolution of phytoplankton related to (the highly selective) fluctuating conditions of the oceans is also reflected in the large MT repertoire that the diatoms possess [47] in comparison to other algae and plants, and high level of gene retention/redundancy (*e.g.*, in dinoflagellates [50], [51]).

Algal derived MTs in diatoms also implicate transporters for inorganic compounds that play key roles in a number of biogeochemical cycles such as sulfur and nitrogen. A recent study demonstrated the importance of nitrate transporters to the survival of diatoms in dark and anoxic conditions [52]. Our findings of red algal derived nitrate transporters in diatoms demonstrate the likely involvement of the red algal endosymbiosis in the establishment of nitrogen assimilation in these taxa. Using a set of strict criteria, our phylogenomic approach provided a conservative estimate of the extent of E/HGT among these MTs in diatom, based on the implicit assumption that units of HGT are whole genes. Therefore, the transfer of gene fragments [53]; *e.g.*, as demonstrated in prokaryotes [54]–[56] and eukaryotes [57], would not have been recovered. In addition, we expect discrepancies to occur between the actual MT repertoire (*i.e.*, the permeome) of diatoms and our current dataset in this work as progress is made in the annotation of these genomes. For instance, a number of MT families are not recovered in both diatom species in our current dataset; *e.g.*, the cation-chloride cotransporter (CCC) and the iron/lead transporter (ILT) are missing in *P. tricornutum*, the copper transporter (Ctr) and the dicarboxylate/amino acid:cation (Na^+^ or H^+^) symporter (DAACS) are missing in *T. pseudonana*. Nevertheless, the rich repertoire of mesophilic red algal genes [27] used in this study provides novel insights into the evolutionary history of these MTs than one previously could arrive at using limited data from the highly reduced gene data from the Cyanidiales.

Although the breadth of the green gene contribution is now better understood, much remains to be clarified about the specific biotic or abiotic selective agents that led to the fixation of so many non-plastid targeted green algal derived MTs in diatoms and other microbial eukaryotes. Given that the red algal secondary endosymbiosis occurred >1 billion years ago [58], the putative green algal endosymbiosis must have occurred earlier. Perhaps this cryptic endosymbiosis, followed by capture of the red algal secondary endosymbiont, provided the other chromalveolate lineages the ability to outcompete other photosynthetic groups such as green algae, in particular prasinophytes (*i.e.*, the “consume and conquer” view of evolution). Pending further investigation and experimental validation, these events could ultimately have led to their dominance in the world's oceans by, for example, being able to deal with changing concentrations of redox-sensitive transition metals over evolutionary timescales. This is particularly relevant given oxidative weathering of continental crusts and its complex interactions with sulphidic oceans that occurred near the time of the eukaryote radiation (*e.g.*, [59]). Apart from the MT data, completed work already suggests that key functions such as photosynthetic performance and protection from oxidative damage in microbial eukaryotes was significantly enhanced by the ancient transfer of green algal derived genes involved in carotenoid biosynthesis [60] and as a central component of the light-harvesting complex superfamily [61]. Studies of green algal derived proteins in diatom with other functions (*e.g.*, biosynthesis, housekeeping, gene expression) may help us identify additional adaptive traits conferred by widespread gene transfer.

## Materials and Methods

### Data

The set of 1,050 MTs from *Thalassiosira pseudonana* (502) and *Phaeodactylum tricornutum* (548) were downloaded from Joint Genome Institute (JGI; ftp://ftp.jgi-psf.org/pub/JGI_data/), based on predictions available at TransportDB (http://www.membranetransport.org/; [24]). Five proteins are redundant (more than one have the reference to the same sequence in GenBank) and were subsequently excluded. In addition, of the 548 *P. tricornutum* proteins, 31 are obsolete (as predicted using earlier models); these proteins were also omitted. The exclusion results in 500 MTs from *T. pseudonana* and 514 MTs from *P. tricornutum* (total of 1,014) for subsequent analysis (Dataset S1). These numbers represent the total predicted MTs (4–5% of each predicted proteomes) in these two diatoms, regardless of gene duplication events.

### Analysis of exclusive phyletic association of genes

We adopted a simplified reciprocal BLAST approach [27] to identify putative homologs for each of these MT proteins based on significant sequence similarity (BLASTP; *e*-value ≤10^−10^). The 1,014 proteins were used to query against a broadly sampled local database (Table S2) that consists all annotated and predicted proteins from GenBank RefSeq release 45 (http://www.ncbi.nlm.nih.gov/RefSeq/) and JGI (ftp://ftp.jgi-psf.org/pub/JGI_data/), respectively, EST data from all available sources of algae and microbial eukaryotes as available from dbEST (http://www.ncbi.nlm.nih.gov/dbEST/) and TBestDB (http://tbestdb.bcm.umontreal.ca/), as well as predicted proteins from the two mesophilic red algae, *Porphyridium cruentum* and *Calliarthron tuberculosum* [27], and the stramenopile *Ectocarpus siliculosus* (https://bioinformatics.psb.ugent.be/gdb/ectocarpus/) [21]. Diatom MTs that have hits only in diatoms and one other taxon based on our BLASTP analysis are inferred as instance of exclusive phyletic association between the two taxa.

### Phylogenomic analysis

In our simplified reciprocal BLASTP approach [27], for each of the top five BLASTP hits (or fewer, if there were fewer than five hits) for a diatom MT protein (query), we generated a list of hits via BLASTP searches against our database. The sequence hits that were found in all of these lists (including the original diatom query) were grouped into a set of putative protein homologs. For each of these sequence sets, we applied two sampling criteria to ensure a reasonable representation of the diverse groupings in a protein set, *i.e.*, ≤5 bacterial subgroups (*e.g.*, Proteobacteria, Actinobacteria, according to the NCBI Taxonomy), and no single species/strain/genome is represented >5 times (these likely represent paralogs). In addition, ≤15 representations from Fungi, and ≤15 representations from Metazoa are included in the protein set. Sequence alignment for each protein set was generated using MUSCLE version 3.8 [62], and refined using GBLOCKS version 0.91b [63] with the options b3 = 20, b4 = 8, and b5 = h to remove divergent, ambiguously aligned blocks (*e.g.,* alignment positions with a high number of gaps that could weaken the phylogenetic signal). For subsequent analysis, we restrict that each protein family (hence alignment) had ≥4 members but were limited to ≤100 members, and that phylogenetically informative sites in each set of aligned protein sequences were ≥50 amino acid positions. We used a maximum likelihood (ML) approach, RAxML [29] for phylogeny reconstruction, under the assumption of WAG amino acid substitution model [64] and a discrete gamma distribution [65], with non-parametric bootstrap of 100. The resulting 879 phylogenies and protein alignments are available at http://dbdata.rutgers.edu/data/diatomMT/. To infer E/HGT in these phylogenies, we restrict our dataset to 697 trees that each consists of ≥3 distinct phyla and ≥30 terminal taxa. We assume phylogenetic artifacts (if present) in these trees to be minimal, although we did not employ any statistical tests to specifically examine the extent of stochastic variations or rate heterogeneity in our data.

### Two-phase approach for phylogeny sorting

The first phase of our approach constitutes the preliminary computational screening of all phylogenies in NEWICK format using a simple text-parsing tool (PERL script available on request). In this screening step, we searched among the 697 trees for strongly supported monophyletic clade (based on non-parametric bootstrap support at ≥70%) between the diatoms and one other taxon (the two target taxa), in which the clade contains ≥2 sequences from each of the target taxon (to minimize effect of missing data). Given the difficulty in reconstructing phylogenies of eukaryotic genes that were impacted by EGT, we additionally allowed interrupting taxa (any lineage other than the two target taxa) to constitute ≤30% of the total number of lineages within the clade. This criterion includes instances of non-exclusive monophyly, *i.e.*, cases where non-lineal history involving more than the two target taxa, *e.g.*, between lineages of diatoms and red algae, as well as the other chromalveolates. The sorted trees are then subjected to the second phase of our approach, during which we manually inspected the sorted trees with respect to gene origins in diatoms. For the ease of manual inspection, trees with ≥3 overlapping proteins are pooled into a single cluster (using Tree Cluster tool in PhyloSort [66]), as they likely represent similar phylogenies within a protein family. All sorted trees in their respective categories and clusters are available at http://dblab.rutgers.edu/home/downloads/. During manual inspection, we used prokaryote lineages as outgroups for tree rooting where possible. If prokaryote taxa were absent from the tree, we used Metazoa as the outgroup root, failing which we used Fungi (both of these latter phyla are opisthokonts). If neither prokaryote nor opisthokont lineages were present in the tree, then we labeled the phylogeny as having an undetermined outgroup and unresolved evolutionary history. Once rooted, we searched the tree for a strongly supported monophyletic clade (bootstrap ≥70%) that consists of two or more diatom lineages and another phylum, as described in the first-phase computational screening.

### Topological comparison using likelihood statistical tests

For each of the 75 RAxML trees in which we observed strongly supported (bootstrap ≥70%) monophyletic clade between stramenopiles and Plantae lineages (using the first-phase computational screening described above), we generated a null hypothesis by requiring the two phyla to be sister to each other within the said clade. The manipulation of tree topologies was done using a pruning approach as implemented in the Analyses of Phylogenetics and Evolution (APE) package in R [67], followed by re-attachment of isolated clades at the designated position of the tree (via text-parsing of NEWICK format) using PERL. We used Tree-Puzzle 5.2 [44] to assess the fit of data (the sequence alignment used to generate the RAxML tree) to each of the RAxML and null hypothesis topologies, using the one-sided KH [40], SH [41], ELW [42] and two-sided KH [43] statistical tests. A rejection of the null hypothesis was inferred at *p*-value ≤0.05.

### Functional annotation

Whereas the putative function for each of these diatom MTs is described in TransportDB, the specific function for each of the diatom MTs is obtained using Blast2GO [68] based on sequence similarity searches (BLASTP, *e*-value ≤10^−6^) against the Swiss-Prot database. The putative functions for proteins that did not have a hit to the Swiss-Prot data are obtained from the specific record of the corresponding (or the most similar) protein sequence of the same diatom species, as available at GenBank (http://www.ncbi.nlm.nih.gov/).

### Prediction of protein target

We used HECTAR (http://www.sb-roscoff.fr/hectar/; [32]) to predict putative subcellular target for each of the 1,014 MT proteins in diatoms based on the presence of transit peptide (specific for chloroplast or mitochondrion in heterokonts), signal peptide, and/or the Type II signal anchor subsequence within the protein, yielding five possible inference for each protein: (a) chloroplast-targeted, (b) mitochondrion-targeted, (c) presence of signal peptide, *i.e.*, these proteins lack the specific transit peptides for chloroplast- or mitochondrion-targeting, (d) presence of a Type II signal anchor, or (e) no N-terminal target peptide found.

## Supporting Information

Figure S1 **Distribution of phyla with exclusive BLASTP hits to diatom MT proteins across the minimum number of hits per query, x ≥2, ≥10, and ≥20.**
(PDF)

Table S1 **The list of transporter families into which the diatom MTs used in this study are classified in TransportDB (http://www.membranetransport.org/).**
(DOCX)

Table S2 **The number of protein sequences in the database that is used for the phylogenomic analysis in this study, based on phyla.**
(DOCX)

Table S3 **Manual inspection of the 399 phylogenetically meaningful trees after initial computational screening.** See also Dataset S1.
(XLSX)

Table S4 **Summary of 1,014 diatom MTs used in this study based on their putative function, gene origin and protein target.**
(XLSX)

Table S5 **Analysis of algal E/HGT in stramenopiles across 75 diatom MTs based on topological comparison against a null hypothesis.**
(XLSX)

Dataset S1 **All 1,014 protein sequences of diatom MTs used in this study.**
(TXT)
