# Supplementary figures and images for: Red and Green Algal Origin of Diatom Membrane Transporters: Insights into Environmental Adaptation and Cell Evolution
Source: PLoS One. 2011 Dec 14;6(12):e29138. doi: 10.1371/journal.pone.0029138 (PMC3237598; doi:10.1371/journal.pone.0029138)

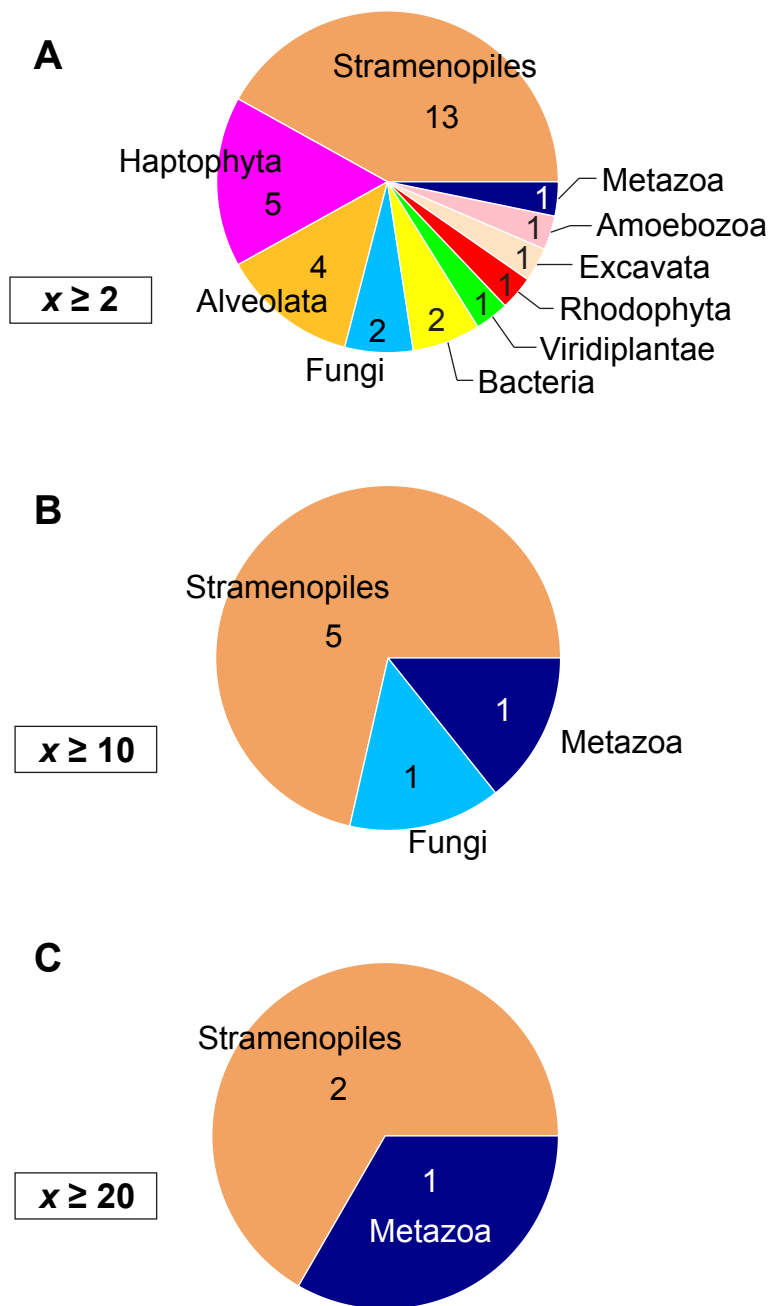

Figure S1

Supplement: Figure S1 — Distribution of phyla with exclusive BLASTP hits to diatom MT proteins across the minimum number of hits per query, x ≥2, ≥10, and ≥20. (PDF) [file pone.0029138.s001.pdf]
