# Supplementary material for: Red and Green Algal Origin of Diatom Membrane Transporters: Insights into Environmental Adaptation and Cell Evolution
Source: PLoS One. 2011 Dec 14;6(12):e29138. doi: 10.1371/journal.pone.0029138 (PMC3237598; doi:10.1371/journal.pone.0029138)
Supplement: Table S1 — The list of transporter families into which the diatom MTs used in this study are classified in TransportDB (http://www.membranetransport.org/). (DOCX) [file pone.0029138.s002.docx]

**Table S1.** The list of transporter families into which the diatom MTs used in this study are classified in TransportDB (<http://www.membranetransport.org/>). Shown for each family is the full name, family abbreviation (ID), number of proteins in *Phaeodactylum tricornutum* (PhT), *Thalassiosira pseudonana* (ThP), and their total. The table is sorted by Family ID in alphabetical order.

| **Full name** | **Family ID** | **PhT** | **ThP** | **Total** |
| --- | --- | --- | --- | --- |
| The ATP:ADP Antiporter (AAA) Family | AAA | 3 | 3 | 6 |
| The Amino Acid/Auxin Permease (AAAP) Family | AAAP | 21 | 17 | 38 |
| The ATP-binding Cassette (ABC) Superfamily | ABC | 51 | 50 | 101 |
| The Anion Exchanger (AE) Family | AE | 4 | 3 | 7 |
| The Auxin Efflux Carrier (AEC) Family | AEC | 1 | 3 | 4 |
| The Amino Acid-Polyamine-Organocation (APC) Family | APC | 3 | 6 | 9 |
| The Ammonia Transporter Channel (Amt) Family | Amt | 8 | 6 | 14 |
| The Annexin (Annexin) Family | Annexin | 2 | 2 | 4 |
| The Arsenite-Antimonite (ArsAB) Efflux Family | ArsAB | 2 | 3 | 5 |
| The Arsenite-Antimonite (ArsB) Efflux Family | ArsB | 2 | 2 | 4 |
| The Bile Acid:Na+ Symporter (BASS) Family | BASS | 8 | 8 | 16 |
| The Betaine/Carnitine/Choline Transporter (BCCT) Family | BCCT | 1 | 1 | 2 |
| The Anion Channel-forming Bestrophin (Bestrophin) Family | Bestrophin | 1 | 0 | 1 |
| The Cation-Chloride Cotransporter (CCC) Family | CCC | 0 | 2 | 2 |
| The Cation Diffusion Facilitator (CDF) Family | CDF | 4 | 4 | 8 |
| The Chloroplast Envelope Protein Translocase (CEPT or Tic-Toc) Family | CEPT | 3 | 2 | 5 |
| The Chromate Ion Transporter (CHR) Family | CHR | 1 | 1 | 2 |
| The Intracellular Chloride Channel (CLIC) Family | CLIC | 2 | 1 | 3 |
| The Monovalent Cation:Proton Antiporter-1 (CPA1) Family | CPA1 | 7 | 6 | 13 |
| The Monovalent Cation:Proton Antiporter-2 (CPA2) Family | CPA2 | 5 | 7 | 12 |
| The Choline Transporter Like (CTL) Family | CTL | 7 | 8 | 15 |
| The Ca^2+^:Cation Antiporter (CaCA) Family | CaCA | 4 | 8 | 12 |
| The Chloride Carrier/Channel (ClC) Family | ClC | 6 | 2 | 8 |
| The Copper Transporter (Ctr) Family | Ctr | 3 | 0 | 3 |
| The Dicarboxylate/Amino Acid:Cation (Na+ or H+) Symporter (DAACS) Family | DAACS | 2 | 0 | 2 |
| The Divalent Anion:Na^+^ Symporter (DASS) Family | DASS | 2 | 2 | 4 |
| The Drug/Metabolite Transporter (DMT) Superfamily | DMT | 47 | 46 | 93 |
| The H^+^- or Na^+^-translocating F-type, V-type and A-type ATPase (F-ATPase) Superfamily | F-ATPase | 27 | 22 | 49 |
| The Folate-Biopterin Transporter (FBT) Family | FBT | 3 | 4 | 7 |
| The Formate-Nitrite Transporter (FNT) Family | FNT | 1 | 1 | 2 |
| The Glutamate-gated Ion Channel (GIC) Family of Neurotransmitter Receptors | GIC | 1 | 4 | 5 |
| The H^+^-translocating Pyrophosphatase (H^+^-PPase) Family | H^+^-PPase | 3 | 2 | 5 |
| The Hydroxy/Aromatic Amino Acid Permease (HAAAP) Family | HAAAP | 2 | 3 | 5 |
| The HlyC/CorC (HCC) Family | HCC | 2 | 3 | 5 |
| The Type II (General) Secretory Pathway (IISP) Family | IISP | 8 | 9 | 17 |
| The Iron/Lead Transporter (ILT) Superfamily | ILT | 0 | 2 | 2 |
| The Inward Rectifier K^+^ Channel (IRK-C) Family | IRK-C | 2 | 1 | 3 |
| The Lysosomal Cystine Transporter (LCT) Family | LCT | 2 | 3 | 5 |
| The Lactate Permease (LctP) Family | LctP | 1 | 1 | 2 |
| The Mitochondrial Carrier (MC) Family | MC | 56 | 52 | 108 |
| The Major Facilitator Superfamily (MFS) | MFS | 62 | 65 | 127 |
| The Major Intrinsic Protein (MIP) Family | MIP | 4 | 2 | 6 |
| The CorA Metal Ion Transporter (MIT) Family | MIT | 3 | 3 | 6 |
| The Multidrug/Oligosaccharidyl-lipid/Polysaccharide (MOP) Flippase Superfamily | MOP | 14 | 16 | 30 |
| The Mitochondrial Protein Translocase (MPT) Family | MPT | 9 | 10 | 19 |
| The Mitochondrial Tricarboxylate Carrier (MTC) Family | MTC | 1 | 1 | 2 |
| The Mg2+ Transporter-E (MgtE) Family | MgtE | 4 | 2 | 6 |
| The Small Conductance Mechanosensitive Ion Channel (MscS) Family | MscS | 8 | 6 | 14 |
| The Nucleobase:Cation Symporter-2 (NCS2) Family | NCS2 | 4 | 1 | 5 |
| The Non-selective Cation Channel-2 (NSCC2) Family | NSCC2 | 1 | 1 | 2 |
| The Neurotransmitter:Sodium Symporter (NSS) Family | NSS | 2 | 0 | 2 |
| The NhaC Na^+^:H^+^ Antiporter (NhaC) Family | NhaC | 1 | 0 | 1 |
| The NhaD Na^+^:H^+^ Antiporter (NhaD) Family | NhaD | 1 | 1 | 2 |
| The Ni^2+^-Co^2+^ Transporter (NiCoT) Family | NiCoT | 1 | 0 | 1 |
| The Metal Ion (Mn^2+^-iron) Transporter (Nramp) Family | Nramp | 0 | 1 | 1 |
| The Cytochrome Oxidase Biogenesis (Oxa1) Family | Oxa1 | 4 | 4 | 8 |
| The P-type ATPase (P-ATPase) Superfamily | P-ATPase | 17 | 17 | 34 |
| The Phosphate:Na^+^ Symporter (PNaS) Family | PNaS | 6 | 3 | 9 |
| The Proton-dependent Oligopeptide Transporter (POT) Family | POT | 2 | 2 | 4 |
| The Peroxisomal Protein Importer (PPI) Family | PPI | 2 | 1 | 3 |
| The Integral Membrane Peroxisomal Protein Importer-2 (PPI2) Family | PPI2 | 1 | 0 | 1 |
| The Inorganic Phosphate Transporter (PiT) Family | PiT | 1 | 2 | 3 |
| The Presenilin ER Ca^2+^ Leak Channel (Presenilin) Family | Presenilin | 2 | 2 | 4 |
| The Resistance-Nodulation-Cell Division (RND) Superfamily | RND | 4 | 4 | 8 |
| The Solute:Sodium Symporter (SSS) Family | SSS | 4 | 2 | 6 |
| The Silicon Transporter (Sit) Family | Sit | 3 | 3 | 6 |
| The Sulfate Permease (SulP) Family | SulP | 5 | 10 | 15 |
| The Telurite-resistance/Dicarboxylate Transporter (TDT) Family | TDT | 0 | 1 | 1 |
| The Transient Receptor Potential Ca2+ Channel (TRP-CC) Family | TRP-CC | 1 | 0 | 1 |
| The Putative 4-Toluene Sulfonate Uptake Permease (TSUP) Family | TSUP | 2 | 2 | 4 |
| The Twin Arginine Targeting (Tat) Family | Tat | 2 | 2 | 4 |
| The Tellurium Ion Resistance (TerC) Family | TerC | 1 | 1 | 2 |
| The Threonine/Serine Exporter (ThrE) Family | ThrE | 1 | 0 | 1 |
| The Chloroplast Envelope Anion Channel-forming Tic110 (Tic110) Family | Tic110 | 1 | 1 | 2 |
| The Urea Transporter (UT) Family | UT | 1 | 2 | 3 |
| The Voltage-gated Ion Channel (VIC) Superfamily | VIC | 15 | 19 | 34 |
| The Vacuolar Iron Transporter (VIT) Family | VIT | 4 | 2 | 6 |
| The YggT or Fanciful K^+^ Uptake-B (FkuB; YggT) Family | YggT | 2 | 2 | 4 |
| The Zinc (Zn^2+^)-Iron (Fe^2+^) Permease (ZIP) Family | ZIP | 10 | 9 | 19 |
| **Total** |  | **514** | **500** | **1,014** |
