# Supplementary material for: Red and Green Algal Origin of Diatom Membrane Transporters: Insights into Environmental Adaptation and Cell Evolution
Source: PLoS One. 2011 Dec 14;6(12):e29138. doi: 10.1371/journal.pone.0029138 (PMC3237598; doi:10.1371/journal.pone.0029138)
Supplement: Table S2 — The number of protein sequences in the database that is used for the phylogenomic analysis in this study, based on phyla. (DOCX) [file pone.0029138.s003.docx]

Table S2. The number of protein sequences in the database that was used for the phylogenomic analysis in this study, based on phyla.

|  | Grouping | Species/Strain | | RefSeq45 | JGI | EST^1^ | Independent^2^ | Total |
| --- | --- | --- | --- | --- | --- | --- | --- | --- |
| **PROKARYOTES** | Archaea | | 124 | 232,382 | 0 | 0 | 0 | 232,382 |
|  | Bact-Actinobacteria | | 313 | 1,098,568 | 0 | 0 | 0 | 1,098,568 |
|  | Bact-Aquificae | | 9 | 17,668 | 0 | 0 | 0 | 17,668 |
|  | Bact-Bacteroidetes/Chlorobi | | 152 | 491,080 | 0 | 0 | 0 | 491,080 |
|  | Bact-Chlamydiae/Verrucomicrobia | | 38 | 79,759 | 0 | 0 | 0 | 79,759 |
|  | Bact-Chloroflexi | | 16 | 55,816 | 0 | 0 | 0 | 55,816 |
|  | Bact-Chrysiogenetes | | 1 | 2,571 | 0 | 0 | 0 | 2,571 |
|  | Bact-Cyanobacteria | | 67 | 221,295 | 0 | 0 | 0 | 221,295 |
|  | Bact-Deferribacteres | | 3 | 7,438 | 0 | 0 | 0 | 7,438 |
|  | Bact-Deinococci/Thermus | | 13 | 28,564 | 0 | 0 | 0 | 28,564 |
|  | Bact-Dictyoglomi | | 2 | 3,656 | 0 | 0 | 0 | 3,656 |
|  | Bact-Elusimicrobia | | 2 | 2,305 | 0 | 0 | 0 | 2,305 |
|  | Bact-Environmental | | 2 | 408 | 0 | 0 | 0 | 408 |
|  | Bact-Fibrobacteres/Acidobacteria | | 7 | 32,953 | 0 | 0 | 0 | 32,953 |
|  | Bact-Firmicutes | | 852 | 2,332,325 | 0 | 0 | 0 | 2,332,325 |
|  | Bact-Fusobacteria | | 26 | 71,012 | 0 | 0 | 0 | 71,012 |
|  | Bact-Gemmatimonadetes | | 1 | 3,935 | 0 | 0 | 0 | 3,935 |
|  | Bact-Nitrospirae | | 3 | 6,366 | 0 | 0 | 0 | 6,366 |
|  | Bact-Planctomycetes | | 7 | 41,568 | 0 | 0 | 0 | 41,568 |
|  | Bact-Proteobacteria | | 1,295 | 4,480,393 | 0 | 0 | 0 | 4,480,393 |
|  | Bact-Spirochaetes | | 44 | 74,482 | 0 | 0 | 0 | 74,482 |
|  | Bact-Synergistetes | | 7 | 15,561 | 0 | 0 | 0 | 15,561 |
|  | Bact-Tenericutes | | 57 | 31,509 | 0 | 0 | 0 | 31,509 |
|  | Bact-Unclassified | | 8 | 14,920 | 0 | 0 | 0 | 14,920 |
|  | Thermotogae | | 12 | 23,420 | 0 | 0 | 0 | 23,420 |
| **EUKARYOTES** | Amoebozoa | | 22 | 30,550 | 12,410 | 138,624 | 0 | 181,584 |
|  | Alveolata | | 68 | 165,903 | 0 | 584,904 | 0 | 750,807 |
|  | Cryptophyta | | 8 | 1,419 | 0 | 40,320 | 0 | 41,739 |
|  | Excavata | | 30 | 134,638 | 0 | 443,424 | 0 | 578,062 |
|  | Haptophyta | | 5 | 140 | 39,124 | 56,868 | 0 | 96,132 |
|  | Opisthokonta-Choanoflagellida | | 4 | 9,203 | 0 | 74,886 | 0 | 84,089 |
|  | Opisthokonta-Fungi | | 192 | 575,573 | 212,456 | 132,168 | 0 | 920,197 |
|  | Opisthokonta-Metazoa | | 2,233 | 1,117,465 | 140,855 | 30,108 | 0 | 1,288,428 |
|  | Opisthokonta-Others | | 4 | 0 | 0 | 46,494 | 0 | 46,494 |
|  | Plantae-Glaucophyta | | 3 | 149 | 0 | 57,696 | 0 | 57,845 |
|  | Plantae-Rhodophyta | | 28 | 1,242 | 0 | 741,132 | 28,975 | 771,349 |
|  | Plantae-Viridiplantae | | 243 | 395,592 | 114,102 | 114,294 | 0 | 623,988 |
|  | Rhizaria | | 5 | 1,211 | 0 | 29,112 | 0 | 30,323 |
|  | Stramenopiles | | 48 | 42,122 | 81,762 | 96,078 | 16,533 | 236,495 |
|  | Vira | | 2,507 | 87,994 | 0 | 0 | 0 | 87,994 |
|  | Others | | 39 | 1,058 | 0 | 0 | 0 | 1,058 |
|  | **Total** | | **8,500** | **11,934,213** | **600,709** | **2,586,108** | **45,508** | **15,166,538** |
| ^1^ The actual numbers of ESTs are the numbers in this column divided by 6 due to six-frame translations.  ^2^ These data represent protein models from *Cyanidioschyzon merolae* and *Calliarthron tuberculosum* (Rhodophyta), and *Ectocarpus siliculosus* (stramenopile). | | | | | | | | |
